# What does my patient's coronary artery calcium score mean? Combining information from the coronary artery calcium score with information from conventional risk factors to estimate coronary heart disease risk

**DOI:** 10.1186/1741-7015-2-31

**Published:** 2004-08-24

**Authors:** Mark J Pletcher, Jeffrey A Tice, Michael Pignone, Charles McCulloch, Tracy Q Callister, Warren S Browner

**Affiliations:** 1 Department of Epidemiology and Biostatistics, University of California, San Francisco 500 Parnassus Ave, MU 420 West, Box 0560, San Francisco, CA 94143-0560, USA; 2 Division of General Internal Medicine, University of California, San Francisco, CA, USA; 3 Division of General Internal Medicine and Clinical Epidemiology, University of North Carolina – Chapel Hill School of Medicine, Chapel Hill, NC, USA; 4 EBT Research Foundation, Nashville, TN, USA; 5 Research Institute, California Pacific Medical Center, San Francisco, CA, USA; 6 Department of Medicine, University of California, San Francisco, CA, USA

## Abstract

**Background:**

The coronary artery calcium (CAC) score is an independent predictor of coronary heart disease. We sought to combine information from the CAC score with information from conventional cardiac risk factors to produce post-test risk estimates, and to determine whether the score may add clinically useful information.

**Methods:**

We measured the independent cross-sectional associations between conventional cardiac risk factors and the CAC score among asymptomatic persons referred for non-contrast electron beam computed tomography. Using the resulting multivariable models and published CAC score-specific relative risk estimates, we estimated post-test coronary heart disease risk in a number of different scenarios.

**Results:**

Among 9341 asymptomatic study participants (age 35–88 years, 40% female), we found that conventional coronary heart disease risk factors including age, male sex, self-reported hypertension, diabetes and high cholesterol were independent predictors of the CAC score, and we used the resulting multivariable models for predicting post-test risk in a variety of scenarios. Our models predicted, for example, that a 60-year-old non-smoking non-diabetic women with hypertension and high cholesterol would have a 47% chance of having a CAC score of zero, reducing her 10-year risk estimate from 15% (per Framingham) to 6–9%; if her score were over 100, however (a 17% chance), her risk estimate would be markedly higher (25–51% in 10 years). In low risk scenarios, the CAC score is very likely to be zero or low, and unlikely to change management.

**Conclusion:**

Combining information from the CAC score with information from conventional risk factors can change assessment of coronary heart disease risk to an extent that may be clinically important, especially when the pre-test 10-year risk estimate is intermediate. The attached spreadsheet makes these calculations easy.

## Background

Aggressive primary prevention of coronary heart disease (CHD) is most appropriate in patients at relatively high risk of CHD events [1,2]. The coronary artery calcium (CAC) score is an independent predictor of coronary heart disease risk [3-7], and therefore may help in deciding how aggressively to pursue cholesterol-lowering, anti-platelet therapy and other primary prevention strategies. To use a given CAC score result, however, one must know how that score compares with the score of an average person of the same sex, age and CHD risk factor profile. A CAC score of 50, for example, may be unusually high for a 40-year-old woman without other CHD risk factors, but unusually low for a 70-year-old man with hypertension. The same score, therefore, affects risk assessment in opposite directions for these two patients. How should a clinician use this CAC score (or any other) when assessing the CHD risk of a more typical patient, say a 60-year-old woman with hypertension and high cholesterol?

To answer this question, we need to know the effects of age, sex and other CHD risk factors on the expected distribution of CAC scores. Several large cross-sectional studies have described the prevalence and extent of CAC among different age/sex groups [6,8-10] without accounting for conventional CHD risk factors that may strongly influence predicted CAC scores. Five previous studies examined how CAC relates to conventional CHD risk factors [11-15]. Only one of these was adequately powered [15], none adequately accounted for the abnormal distribution of CAC scores, and none yielded estimates usable for clinical decision-making.

We identified a large sample of men and women without clinical CHD who presented for electron beam computed tomography scanning. Using questionnaire data collected from these patients about smoking habits and medical history (hypertension, high cholesterol and diabetes), we determined how conventional CHD risk factors, along with age and sex, affect CAC scores. We then developed a method for combining information from conventional risk factors and the CAC score (easy spreadsheet calculator attached), and we present several examples illustrating how that method may be applied in common clinical situations.

## Methods

### Study sample

All persons referred by their physician to an electron beam computed tomography (EBCT) scanning center in Nashville, Tennessee for measurement of coronary artery calcification between May 15, 1995 and December 31, 1997 were eligible for inclusion. Subjects with a history of CHD or complaining currently of any chest pain were excluded, as were subjects for whom CHD risk factor data were incomplete or missing. Only the first CAC score was included for those who received more than one EBCT scan.

### Measurement of coronary heart disease risk factors

Current age, sex and presence of CHD risk factors were elicited by questionnaire from subjects and referring physicians. Each subject was labeled with hypertension, high cholesterol and/or diabetes mellitus if they answered affirmatively to the question, "Has your physician ever told you that you needed medicine for X?", or if their physician confirmed that such a condition was documented in their medical records. Patients were labeled as smokers if they currently smoked or had quit smoking within the preceding 3 months. No direct measurements of blood pressure, lipids or glucose were taken for the purposes of this study.

### Estimation of the 10-year risk of coronary heart disease events

We estimated the 10-year risk of a first CHD event using published mathematical models based on the Framingham study [16]. For this purpose, we assumed that subjects reporting hypertension had systolic blood pressures of 140–160 mmHg and/or diastolic blood pressures of 90–100 mmHg (Stage I hypertension), and that subjects without hypertension had systolic pressures of 120–130 and diastolic pressures of 80–85 mmHg. We also assumed that patients with high cholesterol had low-density lipoprotein (LDL) cholesterol levels of 130–159 mg/dl and high density lipoprotein (HDL) cholesterol levels of 35–44 mg/dl, whereas patients without high cholesterol had LDL cholesterol levels of 100–129 mg/dl and HDL cholesterol levels of 45–49 mg/dl (for men) or 50–59 mg/dl (for women). Smoking and diabetes mellitus were dichotomous variables in both Framingham models [16] and our data set. We then used published model coefficients [16] to estimate the 10-year risk for each patient in our study.

### Measurement of the CAC score

Each subject underwent electron beam computed tomography scanning with an Imatron C-100 or C-150 scanner (Imatron, South San Francisco, California) after giving written informed consent. During a single breath hold, 40 consecutive slices of 3 mm thickness were obtained starting at the level of the carina and proceeding to the level of the diaphragm. Scans were obtained within 100 ms and were electrocardiographically triggered at 60–80% of the R-R interval. Coronary calcification was defined as a plaque of at least 3 consecutive pixels (area = 1.03 mm^2^) with density ≥ 130 Hounsfield units. The CAC score was calculated according to the method described by Agatston [17].

### Statistical analysis

**Figure 1:**
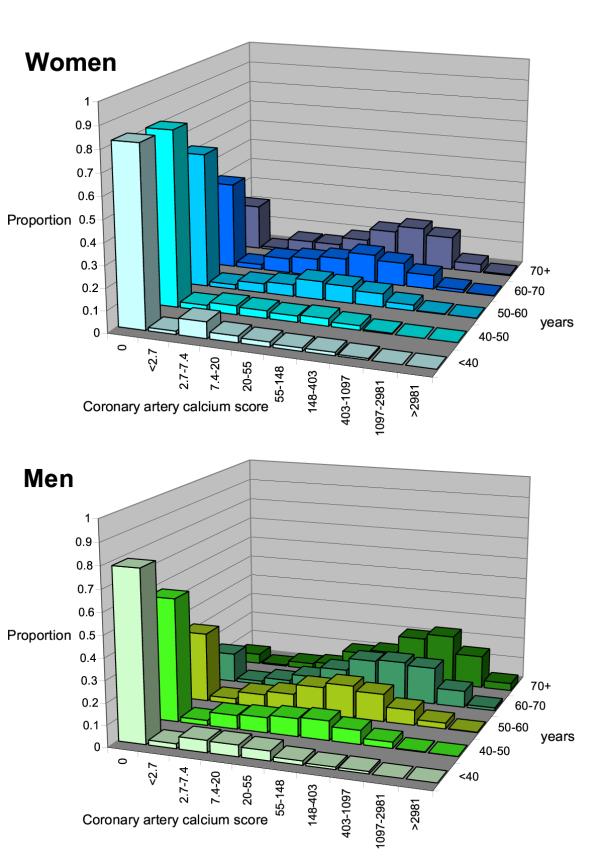
**Distribution of coronary artery calcium scores among men and women, on a logarithmic scale, by age. **Categories chosen for histograms are evenly spaced on a logarithmic scale, corresponding to Ln(CAC) scores of <1, 1–2, 2–3, 3–4, 4–5, 5–6, 6–7, 7–8, and >8. The first bar represents subjects with no detectable CAC, which corresponds to an undefined Ln(CAC) value. CAC – Coronary artery calcium.

We categorized patients according to age and sex, and examined histograms, quantile plots and box plots in each category to evaluate distributional normality. The CAC score is fundamentally not normally distributed because of the large percentage of zero measurements, and hence is not amenable to a normalizing transformation, as noted by others [13]. We also considered a censored normal distribution, which would have allowed a one-step Tobit regression analysis. However, even after square- and cube-root transformations, the zero scores were distributed in a manner inconsistent with the Tobit regression model. After exclusion of zero values, however, the log-transformed CAC score was approximately normally distributed (Figure 1).

This led us naturally to a two-stage modeling approach. We first applied logistic regression to model the probability of a non-zero score, and then used linear regression to model the actual CAC score, log-transformed, for the subset of patients with non-zero values. Using this methodology, we assessed the independent effects of CHD risk factors on both the presence and extent of CAC.

We considered three sets of predictors: 1) age and sex, 2) age, sex, hypertension, high cholesterol, smoking, and diabetes, and 3) the Framingham 10-year CHD risk estimate. We examined whether the effects of age were linear (as opposed to J-shaped, for example) by testing a quadratic term in the model containing only age and sex. We evaluated the ability of each logistic model to discriminate subjects at high and low risk for CAC using the C-statistic, and estimated the proportion of variability in the extent of CAC explained in each linear regression model using the adjusted-R^2 ^statistic.

Finally, we used coefficients, intercepts and residual variance from logistic and linear models to estimate the probability that the CAC score of an individual with known risk factors would fall into each of four standard CAC score categories (0, 1–100, 101–400, and >400). We estimated these probabilities, using models containing the 10-year risk estimate as the only predictor, for a range of 10-year risk estimates. We also estimated these probabilities, using models with all CHD risk factor predictors, for the specific clinical scenario described in the Introduction (a 60-year-old woman with hypertension and high cholesterol) and for several other scenarios. We compared the actual distribution of CAC scores among 58–62-year-old women with hypertension and high cholesterol in our sample (n = 130) with predictions from 1) our two-stage model, 2) a one-stage model using Ln(CAC score + 1) as a continuous outcome in a linear regression model, and 3) a one-stage model using a censored normal distribution of cube-root transformed CAC scores (a Tobit regression model). This comparison was made both graphically and statistically, using X^2 ^tests with 3 degrees of freedom to compare the expected frequencies based on each model with the observed frequencies. Lower p values, in this case, indicate a poorer fit of the model to the observed data. All statistical analyses were performed with Stata 7.0 (College Station, Texas).

### Combining information from conventional risk factors and the CAC score

First, we calculated the Framingham 10-year CHD risk estimate (and corresponding 1-year risk estimate assuming an equal event rate each year) according to published models [16]. Next, we calculated the probability, as described above, that that individual's CAC score would fall into each one of four standard CAC score categories [15,18,19] (0, 1–100, 101–400, and >400). We obtained risk factor-adjusted relative risk (RR) estimates from a meta-analysis [7] comparing the risk of a CHD event among persons with CAC scores of 1–100 (RR = 2.1), 101–400 (RR = 5.4) and <400 (RR = 10) to the risk in a person with a CAC score of zero. The analysis was repeated using more conservative estimates from the same paper: RR = 1.7 (for CAC 1–100), RR = 3.0 (for CAC 101–400), and RR = 4.3 (for CAC>400). The post-test CHD risk estimates for each CAC score category were then calculated algebraically by assuming that the overall 1-year CHD risk estimate represents an average of the 1-year risk estimates from the four CAC score categories, weighted by the probabilities that an individual's score would fall into each category. A spreadsheet that automates these calculations is attached.

## Results

### Study population

We identified 9341 persons without chest pain or a history of CHD presenting for their first EBCT scan between 4/15/95 and 12/31/97. Our sample was mostly middle-aged, but included persons as young as 35 years and as old as 88 years of age. Forty percent were women. The proportion with cardiac risk factors was high, though only 9% were diabetic (Table 1). Framingham 10-year CHD risk estimates ranged widely, mostly dependent on age, but most were between 7% and 15%.

### Coronary artery calcium score distributions

Coronary artery calcium scores ranged from 0 to 4058. The mean score (± standard deviation) was 135 (± 377), and the median was 4 (25^th^–75^th ^percentile: 0 – 87). The prevalence of zero scores ranged from 80% among women younger than 50 years to 5% among men 70 years old or older. After excluding zero scores, log-transformed CAC scores were approximately normally distributed, and appeared to be strongly associated with age and sex (Figure 1).

### Predictors of the presence and extent of coronary artery calcification

Age and sex were strong predictors of the presence of CAC in logistic regression models (Table 2). There was no evidence that the effects of age were non-linear (i.e. J- or U-shaped) (p-value = 0.32 for a quadratic age term). Conventional CHD risk factors were also independent predictors of the presence of CAC (p < 0.001 in all cases). The logistic model with age, sex and all CHD risk factors produced the most accurate model (C-statistic = 0.78). The Framingham 10-year CHD risk estimate was also a very strong predictor of coronary artery calcification, though the model containing the 10-year risk estimate as the only predictor was slightly less accurate (C-statistic = 0.74).

Among patients with non-zero CAC scores, age and sex remained strong predictors of the extent of coronary artery calcification, as measured by the Ln(CAC score) (Table 3). Again, the effects of age appeared to be linear (p = 0.16 for the quadratic age term). All conventional CHD risk factors remained statistically significant predictors of the extent of coronary artery calcification (p < 0.001 for all predictors except high cholesterol at p = 0.004). Again, the Framingham 10-year CHD risk estimate was a very strong predictor of the extent of calcification, though when used alone in a model, it explained somewhat less of the variance (R^2 ^= 0.11) than the full model (R^2 ^= 0.17).

### Coronary artery calcium distribution predictions

Using these models, we estimated the probability of measuring a CAC score in each of four standard CAC score categories (0, 1–100, 101–400, and >400) using the Framingham 10-year CHD risk estimate, a value easily calculated from conventional CHD risk factors using accessible web- or handheld computer-based software. These probabilities ranged widely based on the value of the 10-year risk estimate, with the probability of measuring a zero CAC score going from 75% (at a 10-year risk of 2.5%) to 13% (at a 10-year risk of 25%) (Table 4).

### Risk integration example

Using the case example presented in the Background section, we calculated that a 60-year-old woman with Stage I hypertension (140/90 mmHg) and high cholesterol (LDL cholesterol = 155 mg/dl, HDL cholesterol = 40 mg/dl) will have a 15% risk of suffering a CHD event in 10 years, according to the Framingham equation. If this women undergoes EBCT scanning, our models predict a 47% chance that her CAC score will be zero, a 36% chance that it will be between 1–100, a 12% chance that it will be between 101–400, and a 5% chance that it will be greater than 400. By integrating this information with previously published relative risk estimates (see Additional File 1), we estimate her 10-year CHD risk to be as low as 6% (if her CAC score is 0), or as high as 51% (if her CAC score is >400). These estimates are only moderately sensitive to variation in the relative risk assumptions (Table 5), and may be easily calculated in any clinical scenario in which CHD risk factor data is available; see Table 5 for several other examples.

### Comparing predictions from different modeling strategies

**Figure 2:**
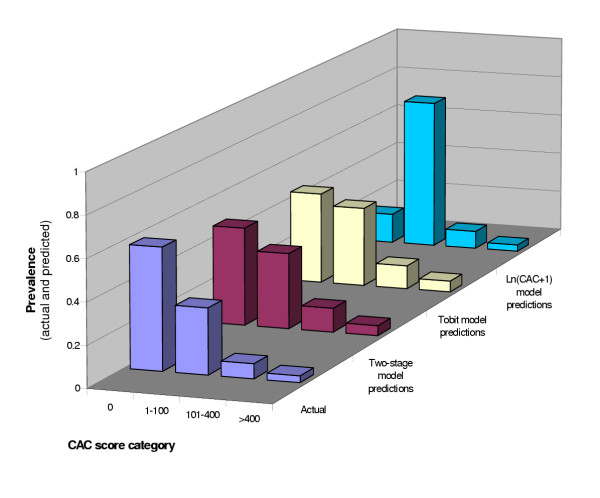
**Comparison between actual and predicted CAC score distributions among a subset of the study population using three different modeling strategies. **Actual prevalence measurements were from the 58- to 62-year-old non-smoking women in our study sample with hypertension, high cholesterol level, and no diabetes (n = 127). The "two-stage model predictions" use the coefficients presented in Tables 2 and 3 (the full model). The Ln(CAC+1) model predictions are from a linear regression model including all conventional CHD risk factors using Ln(CAC score +1) as a continuous outcome in a one-step modeling process (coefficients not presented). The Tobit model uses the cube-root of the CAC score as a continuous outcome for linear regression analysis, but assumes that scores at or below zero have been censored (coefficients not presented). P-values refer to a X^2 ^test with 3 degrees of freedom comparing the expected frequencies based on each model with the observed frequencies. Lower p-values indicate a poorer model fit. CAC – Coronary artery calcium; CHD – Coronary heart disease; Ln – Natural logarithm.

Our strategy outperformed two other modeling strategies in predicting the actual CAC distribution among the 58–62-year-old non-smoking non-diabetic women with hypertension and high cholesterol in our study sample (n = 127) (Figure 2). The one-stage regression model using Ln(CAC score +1) as the outcome, which has been utilized extensively in previous research [11,12,14,20], performed particularly poorly.

## Discussion

In this article, we present a clinically useful method of combining information from the CAC score with pre-test coronary risk estimates. To fully appreciate the utility of this analysis, it may be worthwhile to discuss the example from the Background section further. According to current guidelines, this 60-year-old woman, whose 10-year CHD risk estimate is about 15%, should receive both aspirin and cholesterol-lowering drug therapy, aiming for a goal LDL cholesterol of 130 mg/dl [1,2]. After measuring her CAC score, however, there is a good chance (64%) that our recommendations would change. If her CAC score were zero (47% chance), our estimate of her 10-year CHD risk would be approximately halved (6–9%). Given this information, we would continue to recommend a healthy diet and exercise, but might decide that cholesterol-lowering medication is unnecessary [1], and that the benefits of aspirin in terms of CHD prevention do not outweigh the risk of hemorrhagic stroke associated with aspirin use [2]. On the other hand, if her CAC score were over 100 (17% chance), our estimate of her CHD risk would be approximately doubled (25–31% if CAC score = 101–400) or tripled (34–51% if CAC score > 400). In such a case, we would certainly recommend both aspirin [2] and cholesterol-lowering medication [1] and would probably aim for a more aggressive LDL cholesterol goal of < 100 mg/dl [1]. The probability that her treatment plan would be altered by measurement of her CAC score, therefore, is approximately 64% (the probability that her score is either 0 or >100 = 47% + 17%), indicating likely usefulness of the test in this situation.

The third and fourth clinical scenarios presented in Table 5, on the other hand, provide examples where the test is unlikely to change management. The 40-year-old woman who smokes, for example, has a very low pre-test 10-year CHD risk (3%). It is very likely her CAC score will be zero (89%) or less than 100 (10%), in which case her post-test 10-year CHD risk will still be low (≤ 5%) and her management would not change. The 80-year-old man with high cholesterol has a high pre-test 10-year CHD risk (26%) and a high probability of having a high CAC score (70% will have a score > 100), in which case his post-test 10-year CHD risk would remain over 20% and his management would have to remain aggressive. In these cases, and others in which the risk factor profile indicates very low or very high pre-test risk, the test is not likely to provide useful results, and the clinician might decide not to order the test. We have provided a simple spreadsheet (see Additional File 1) that may be used by readers of this article to replicate these analyses and apply our models to other clinical scenarios.

While others have proposed similar Bayesian approaches to use of the CAC score for coronary risk prediction [6,21-24], ours has advantages. Previous approaches do generally take into account the pre-test probability of coronary heart disease, but none consider the expected distribution of CAC scores in the tested population after adjustment for conventional CHD risk factors. Raggi et al advocate use of an age- and sex-adjusted calcium score percentile, but this ignores both persons with zero scores and the strong effects of other risk factors such as hypertension and hypercholesterolemia [6]. Some approaches use only sensitivity and specificity from dichotomized CAC score cutoffs [21,23], and others use CAC score-specific relative risks generated from a single study population [6,24]. Only two provide actual post-test risk estimates for specific clinical situations [23,24]. Our approach takes into account the pre-test coronary risk, the expected distribution of CAC scores adjusted for all conventional CHD risk factors, and summary adjusted relative risks from a recent meta-analysis, and provides clinically relevant post-test risk estimates that may be directly useful to primary care physicians, cardiologists and patients as they decide whether or not to take medications for primary prevention of CHD.

This analysis confirms that conventional risk factors for CHD (hypertension, diabetes, smoking and high cholesterol, as well as increasing age and male sex) are independent predictors of coronary artery calcification. This finding is consistent with previous studies [11-15]. We also present expected CAC score distributions for a variety of clinical situations, which are not easily calculated from other studies, via Tables 4 and 5 and the attached spreadsheet calculator. Our finding that high cholesterol was less strongly associated with the extent of CAC than other CHD risk factors is consistent with the other large study addressing this issue [15], and perhaps reflects effective medical treatment for hypercholesterolemia. Male sex was a very strong predictor of the presence and extent of CAC – women with the same CHD risk factor profile would be expected to develop CAC approximately 12 years later than men, and remain approximately 11 years behind men in the extent of their calcification.

Finally, our analysis provides a guide for how to use the CAC score as a surrogate outcome when studying causes of coronary artery disease (a widely used study design [25-27]). The central problem with this approach is the fundamentally non-normal distribution of CAC scores, which makes parametric statistic testing (including both simple t-tests and multivariable linear regression) invalid. In dealing with this issue, some researchers have used the Ln(CAC score +1) as an outcome in linear regression analyses [11,12,14,20]. This approach is not ideal, as the Ln(CAC score +1) is still grossly non-normal – there are too many zero scores. Adding 1 to the CAC score makes the log-transformation possible (yielding zeroes instead of negative infinity), but it does not solve the distributional problem, and leads to predictions that misrepresent actual CAC score distributions (Figure 2). This observation has led others to present only non-parametric percentile data without multivariable modeling [6,8-10], but this approach does not allow adjustment for conventional CHD risk factors that we have shown are strong predictors of the CAC score. One other group used ordinal logistic regression analysis to analyze CAC scores categorized into four ordinal categories (quartiles in their study sample) [13]. While such an approach does allow multivariable modeling with ordinal logistic regression, it does not take full advantage of the continuous nature of the CAC score and may blur the important distinction between zero and non-zero scores. Our analysis suggests that a two-step approach (using first logistic regression to model the risk of having a non-zero score, then linear regression of log-transformed non-zero CAC scores to model the extent of coronary calcification) will allow multivariable analysis of the interval data provided by the CAC score without violating the basic assumptions of parametric statistics.

Our analysis has a number of limitations, perhaps the most important being a lack of clinical detail about participants. While we had information about conventional risk factors (hypertension, high cholesterol, diabetes mellitus and tobacco use), the data were only available from a questionnaire, and were not confirmed by direct measurement. Only dichotomous indicators of such conditions were used. Furthermore, other conditions and indicators of high CHD risk such as family history of CHD, obesity, physical activity, income, education, and levels of C-reactive protein, triglycerides and Lp(a), for example, were unavailable. Whether such factors are important predictors of the presence and extent of coronary artery calcification is unknown. On the other hand, CHD risk assessment is often based on the same type of limited information we had available on each of our patients, so the models we present are perhaps more easily applicable to common clinical situations than models based on more detailed clinical data. Furthermore, a historical indicator of past exposure to high blood pressure or high cholesterol, as we had access to in this study, may actually be more useful as a predictor of CAC than treated blood pressure measured at one point in time. Another important limitation of this study is our lack of data on race/ethnicity – our results may not apply to all ethnic groups. Finally, our data are limited in application to CAC scores measured by electron beam computed tomography with 3 mm slice thickness and the described protocol. While CAC scores measured by the latest spiral computed tomography scanners appear to be similar to those generated by electron beam computed tomography [28], we cannot guarantee that our results apply to such scores. Our models should be applied to other similar cohorts for validation, and also applied in cohorts that include different racial/ethnic groups and different ways of measuring the CAC score before being used in these clinical situations.

## Conclusions

The Clinical Research Roundtable at the Institute of Medicine has identified translation of clinical research findings into improvements in medical care as the "next scientific frontier" [29]. While our analysis has some limitations, it provides methodology that will directly assist in the translation of research into practice. Our models, once validated, can be used directly by patients and clinicians to decide when it might be useful to order this potentially expensive test, and what to do with the results.

## Competing interests

MP has received speaking and consulting fees from Bayer.

## Authors' contributions

MJP conceived the idea for the study, performed the analysis and drafted the manuscript. JAT and MP helped design and interpret the analysis. CM provided statistical guidance and interpretation. TQC recruited the patients and collected the data. WSB provided senior guidance in all aspects. All authors reviewed and commented on multiple drafts of the manuscript and approved the final draft.

## Pre-publication history

The pre-publication history for this paper can be accessed here:



## Supplementary Material

Additional File 1 This spreadsheet is used for combining information from conventional risk factors and the coronary artery calcium score to estimate coronary heart disease risk in an individual patient. Step 1: Enter your patient's clinical information (the red numbers). Step 2: Choose an assumption about the coronary artery calcium score relative risks (optimistic or conservative). Step 3: Find the following results: 1) "Pre-test" 10-year risk of coronary heart disease (CHD) based on Framingham equations; 2) The probability of having a coronary artery calcium (CAC) score that falls within 4 standard CAC score categories; and 3) The "post-test" 10-year risk of CHD for each CAC score category. Step 4: Use the results to interpret a CAC score, or to decide whether or not to order a coronary artery calcium scan. If a score that would change your management is unlikely to occur, it may not be worth the money. Click here for file

## Figures and Tables

**Table 1:** Characteristics of 9341 patients meeting inclusion criteria

Characteristic	N (%) or mean ± SD
Age (years)	54 ± 10 years
Women	3782 (40%)
Hypertension*	4069 (44%)
High cholesterol*	5847 (63%)
Diabetes mellitus*	807 (9%)
Smoking†	3679 (39%)
Framingham 10-year risk estimate	
- range	1.0% – 74%
- median, 25%–75%	11%, 7.0% – 15%

* – Per self report. Patients were asked whether they were under medical treatment for "X".

† – Current, or quit within the past six months

SD – Standard deviation

**Table 2:** Predictors of the presence of coronary artery calcium, in three logistic regression models

Modeling approach	Model results		
Predictors	Odds ratio (95% CI)	p-value	C-statistic

Age and sex only*			
- Age, per 10 years	2.83 (2.67 – 2.99)	<0.001	0.76
- Male sex	3.60 (3.26 – 3.96)	<0.001	
All CHD risk factors*			
- Age, per 10 years	2.78 (2.62 – 2.94)	<0.001	0.78
- Male sex	3.67 (3.31 – 4.06)	<0.001	
- Hypertension	1.51 (1.37 – 1.66)	<0.001	
- Diabetes mellitus	1.85 (1.55 – 2.21)	<0.001	
- High cholesterol	1.40 (1.27 – 1.54)	<0.001	
- Smoking	1.71 (1.56 – 1.89)	<0.001	
Estimated 10-year risk of CHD†, only*			
- 10-year risk, per 5% increase in the Framingham 10-year CHD risk estimate	1.96 (1.88 – 2.04)	<0.001	0.74

* – Intercepts (on a log-odds scale) were -6.20, -6.76, and -1.44 for each model, respectively.

† – 10-year risk of CHD estimated according to Framingham equations [16]; for assumptions used, see Methods.

CHD – Coronary heart disease; CI – Confidence interval.

**Table 3:** Predictors of the extent of coronary artery calcium, as measured by log-transformed non-zero coronary artery calcium scores, in three linear regression models.

Model approach	Model results			
Predictors	Coefficients (95% CI)	Corresponding percent increase in natural CAC scores*	p-values	Adjusted R^2^

Age and sex only†				
- Age, per 10 years	0.68 (0.63 – 0.73)	97% (88 – 107%)	<0.001	0.14
- Male sex	0.72 (0.61 – 0.82)	105% (85 – 127%)	<0.001	
All CHD risk factors†				
- Age, per 10 years	0.69 (0.64 – 0.73)	99% (89 – 109%)	<0.001	0.17
- Male sex	0.73 (0.63 – 0.83)	108% (88 – 130%)	<0.001	
- Hypertension	0.23 (0.14 – 0.32)	26% (14 – 38%)	<0.001	
- Diabetes mellitus	0.48 (0.33 – 0.62)	61% (40 – 88%)	<0.001	
- High cholesterol	0.15 (0.05 – 0.24)	16% (4.8 – 28%)	0.004	
- Smoking	0.45 (0.35 – 0.54)	56% (42 – 71%)	<0.001	
Estimated 10-year risk of CHD‡, only†				
- 10-year risk, per 5% increase	0.34 (0.31 – 0.36)	40% (36 – 44%)	<0.001	0.11

* – The percent increase in the natural (non-transformed) CAC score associated with each predictor is calculated by exponentiating the regression coefficient from the linear regression model (when the dependent variable is log-transformed), and subtracting 1.

† – Intercepts were -0.181, -0.705, and 3.17 for each model respectively. The standard deviations of the residuals were 1.682, 1.653, and 1.707.

‡ – 10-year risk of CHD estimated according to Framingham equations [16]; for assumptions used, see Methods.

CAC – Coronary artery calcium; CI – Confidence interval; CHD – Coronary heart disease.

**Table 4:** Estimated prevalence of a coronary artery calcium score in each of four standard categories, depending on the Framingham estimated 10-year risk of coronary heart disease events.

Framingham 10-year CHD risk estimate*	Estimated prevalence of a CAC score in the given range†, %			
	
	0	1–100	101–400	>400
2.5%	75	19	4	1
5.0%	68	23	6	2
7.5%	61	28	8	3
10.0%	52	32	11	5
12.5%	44	36	13	7
15.0%	36	38	17	9
17.5%	29	40	19	12
20.0%	22	41	22	15
22.5%	17	40	25	18
25.0%	13	39	27	22

* – 10-year risk of CHD events estimated according to equations derived from the Framingham study[16]. For assumptions, see Methods.

† – Proportions of subjects in each given CAC score category were estimated by a two step process: 1) Logistic regression to predict the presence of CAC according to estimated 10-year risk (see Table 2 for model coefficients), and 2) Linear regression to predict the extent of CAC, as measured by the natural log-transformed CAC score (see Table 3 for model coefficients). Other values required for this calculation were the logistic regression constant (-1.44), the linear regression constant (3.17), and the standard deviation of the residuals after linear regression (1.707).

CAC – Coronary artery calcium; CHD – Coronary heart disease.

**Table 5:** Examples of how to use the coronary artery calcium score to refine risk estimates.

Clinical scenario	Pre-test 10-year CHD risk estimate*	CAC score category	Proportion of CAC scores falling within the given category†	Post-test 10-year risk estimate for each CAC score category‡	
				
				Conservative§	Optimistic§
60-year-old woman with hypertension and high cholesterol	15%	0:	0.47	9%	6%
		1–100:	0.36	15%	13%
		101–400:	0.12	25%	31%
		>400:	0.05	34%	51%
50-year-old man without other CHD risk factors	6%	0:	0.59	4%	3%
		1–100:	0.31	7%	6%
		101–400:	0.07	11%	15%
		>400:	0.03	16%	27%
40-year-old woman who smokes	3%	0:	0.89	2%	2%
		1–100:	0.10	4%	5%
		101–400:	0.01	7%	12%
		>400:	0.00	10%	22%
80-year-old man with high cholesterol	26%	0:	0.05	9%	5%
		1–100:	0.25	15%	10%
		101–400:	0.30	26%	23%
		>400:	0.40	35%	39%

* – From published Framingham equations [16].

† – These probabilities are calculated using regression equations presented in Tables 2 and 3 of this paper (full models with all predictors). See Methods for details.

‡ – Post-test risk estimates are calculated by assuming that the pre-test 10-year CHD risk estimate represents an average of persons with different CAC scores, weighted by the probability of having a CAC score in each category. The risk in each category is calculated algebraically using relative risk estimates§ from a recent meta-analysis [7] (see Additional File 1).

§-"Conservative" and "Optimistic" refer to assumptions made in a recent meta-analysis that attempted to quantify the value of CAC scores in predicting CHD events, independent of other CHD risk factors [7]. With conservative assumptions, relative risks associated with different CAC score categories were 1.7 (for CAC = 1–100), 3.0 (for CAC = 101–400) and 4.3 (for CAC>400) compared with a CAC score of zero. With optimistic assumptions, the corresponding relative risks were 2.1, 5.4, and 10.3.

CHD – Coronary heart disease; CAC score – Coronary artery calcification score.
